# Simulated risk of root and neurovascular bundle damage during miniscrew insertion in the anterior palate without radiological data

**DOI:** 10.3389/fdmed.2026.1729079

**Published:** 2026-03-09

**Authors:** Antonino Lo Giudice, Vincenzo Ronsivalle, Alessandro Polizzi, Orazio Bennici, Giuseppe Palazzo

**Affiliations:** 1 Department of General Surgery and Medical-Surgical Specialties, University of Catania, Catania, Italy; 2 Private Practice, Catania, Italy

**Keywords:** digital orthodontics, miniscrews, skeletal anchorage, surgical guide, TADs

## Abstract

**Objective:**

This study aimed to explore the risk of potential root and nerve damage in planning the insertion of paramedian miniscrews without radiological information.

**Study design:**

The study included CBCT and intra-oral scan (IOS) records of 60 subjects (28 males, 32 females) featuring normal transverse palate dimension (Group A = 30 subjects, mean age 20.5 ± 4.7) and skeletal maxillary constriction (Group B = 30 subjects, mean age 21.9 ± 5.1). Two miniscrews (Spider Screws Regular Plus Konic - HDC Srl, Vicenza, Italy), featuring 2 mm in diameter and 9 mm in length were virtually inserted using only the.stl IOS file following established clinical guidelines (T-Zone). Specific linear measurements were registered to calculate the distance between the miniscrews and both incisors’ roots and the naso-palatine duct after integrating CBCT images; same measurements were performed after adjusting the position with the aid of CBCT. All data were analyzed for comparison between both procedures and groups.

**Results:**

Safe distances were recorded between miniscrews and incisors’ roots in both groups (*p* > 0.05). The distances from the nasopalatine duct were significantly closer (and in few cases risky) in group B and required significant adjustment using CBCT (*p* < 0.05).

**Conclusion:**

The present findings would discourage planning or inserting miniscrews in the paramedian region without radiological information (CBCT) in subjects with skeletal maxillary constricted.

## 1 Introduction

Orthodontic miniscrews are widely used to reinforce anchorage and simplify complex orthodontic biomechanics. They have expanded treatment options, particularly in cases where poor patient compliance or residual skeletal growth may affect outcomes (1). Among extra-alveolar sites, the anterior palatal bone is recognized as favorable for miniscrew insertion because it provides sufficient bone depth and two cortical layers, ensuring primary stability and high success rates (2–5). Optimal sites are located within the T-shaped region, which includes the anterior paramedian area and the median suture area (6). Implants in this region demonstrate high stability, with no significant differences between median and paramedian placements (7). The paramedian area is also versatile, offering anchorage for orthodontic or orthopedic appliances that support mechanics in sagittal, transverse, and vertical dimensions (8). Furthermore, the same anchorage can be adjusted during treatment to modify biomechanics (9).

Direct insertion of miniscrews into the anterior palate is considered a secure procedure (10) due to the relative distance from fragile anatomical structures (11). Nevertheless, root damage and transient loss of pulp sensibility in maxillary anterior teeth have been reported when miniscrews are misplaced beyond the T-zone (second rugae) (12, 13). Other common complications include pain, soft- and hard-tissue inflammation, and hypertrophy of palatal tissues surrounding the miniscrew (13). The use of a surgical guide is recommended to improve control over miniscrew inclination and parallelism, facilitating appliance placement and reducing the risk of bone trauma (14). Guided systems also allow precise localization of the nasal cortical bone and orientation of the miniscrew to achieve bi-cortical anchorage (15).

Surgical guides can be designed using commercially available software, based on a lateral cephalogram (16, 17) or a CBCT scan registered with a digital intraoral scan (IOS) (18). CBCT is reserved for cases requiring detailed anatomical analysis, such as severe maxillary constriction or impacted/supernumerary teeth (19). Conversely, a lateral cephalogram is recommended as a routine tool because it is already part of standard orthodontic records, avoiding unnecessary radiation exposure (17, 20). Dynamic guided surgery is also emerging, combining CBCT guidance, real-time instrument tracking, and the ability to adjust position and inclination during insertion (21). Despite these advances, miniscrews are often inserted in the paramedian region without a surgical guide (16), mainly because direct insertion is faster and avoids investment in software or 3D printing. This highlights the need for more comprehensive data on the risk of injury to sensitive structures in the paramedian area without radiological support.

In this regard, the present study aimed to evaluate the risk of root and nerve damage during miniscrew insertion in the paramedian area, with and without radiological information. After simulating insertion using only the patient IOS, the distances between the digital miniscrews and sensitive structures were measured and compared with distances obtained from ideal placement using CBCT. The null hypothesis was that no significant differences exist between measurements obtained with the two methods.

## 2 Materials and methods

The present investigation was carried out in accordance with the Helsinki Declaration on medical protocols and ethics and received approval from the Institutional Ethical Committee of the University of Catania (protocol n. 119/2020/po-A.Q.A.M.DI.). The study included CBCT and intra-oral scan (IOS) records of 60 subjects (28 males, 32 females) with an average age of 21.2 ± 4.85 years, retrospectively retrieved from a broader sample of two private Orthodontic Practices in Catania, Italy. Reasons for CBCT and IOS acquisitions were: 1) planning skeletal anchorage for maxillary expansion in subjects with transverse maxillary deficiency (Group A = 30 subjects, mean age 20.5 ± 4.7) and 2) pre-surgical evaluation of upper third molars before orthodontic treatment (Group B = 30 subjects, mean age 21.9 ± 5.1). Transverse maxillary deficiency was diagnosed based on clinical evidence of a constricted palatal morphology associated with posterior crossbite involving at least two teeth or with a normal transverse relationship resulting from dentoalveolar compensation (accentuated Wilson's Curve) (22). Inclusion criteria were: subjects with skeletal class I (ANB = 2° ± 2°) or skeletal class II (ANB = ≥ 4°), high-quality images without artifacts. Exclusion criteria included previous orthodontic treatment, impacted or supernumerary teeth, tooth agenesis, systemic disease, syndromic conditions, and craniofacial anomalies. Table 1 describes the demographic characteristics of the study sample. Patients were scanned with: 1) the same CBCT machine i-CAT (Imaging Sciences International, Hartfield, PA, 120 kVp, 23.87 mAS, 8.9-second exposure time, large FOV, 0.3-mm voxel size) and data sets images were saved in Digital Imaging and Communications in Medicine (DICOM); 2) the same IOS (Carestream C3600 - Carestream Health Inc., Rochester, New York, USA) and were exported in standard tessellation language (STL) file format. Both files were anonymized to protect patients' data.

**Table 1:** Demography and clinical characteristics of the sample of the study.

Sample	Total (*n* = 30)	Group A (*n* = 15)	Group B (*n* = 15)	*p* value
Characteristics	Mean/SD	Mean/SD	Mean/SD	
Mean age	21.2 (± 4.8)	20.5 (± 4.7)	21.86 (± 5.1)	0.461
Gender				0.712
Male	28	15	13	
Female	32	15	17	
CVMS stage				0.098
Stage IV	37	16	20	
Stage V	23	14	9	

SD, standard deviation; CVMS, Skeletal Maturity Index according to the Cervical Vertebral Maturation Method.

*P*-value for comparison of group means by t-test or differences in proportion calculated by chi-square test.

### 2.1 Preliminary measurements of the miniscrews and components

Because the native STL file of the miniscrews was unavailable, physical measurements of the miniscrews and guided insertion components were obtained using a digital caliper (14). These measurements were used to generate a customized digital replica of the Spider Screw Regular Plus Konic (HDC Srl, Vicenza, Italy), with a diameter of 2 mm and a length of 9 mm, using Blue Sky Plan* software (Blue Sky Bio, version 4.7). The following parameters were recorded: screw body length, apical body diameter, occlusal body diameter, intraoral head length, and intraoral head diameter.

### 2.2 Digital planning

Each patient's intraoral scan (IOS) and CBCT scans were imported into Blue Sky Plan* (Blue Sky Bio, version 4.7), a certified software commonly utilized in restorative implant dentistry. This software facilitates an initial point-based superimposition of IOS and CBCT, followed by final registration using the best-fit algorithm (23). The “customize implant” function was selected to generate a virtual equivalent of the miniscrews, while the equivalent abutment represented the extra-alveolar screw head. This process was performed using the measurements recorded in the preliminary step. Conceptually, the orthodontic miniscrew can be considered as a monophasic temporary implant with an intraosseous portion, an intramucosal neck, and an intraoral portion (miniscrew head). The intraosseous portion and gingival neck constitute the screw body, while the head of the miniscrew serves as the abutment.

**Figure 1:**
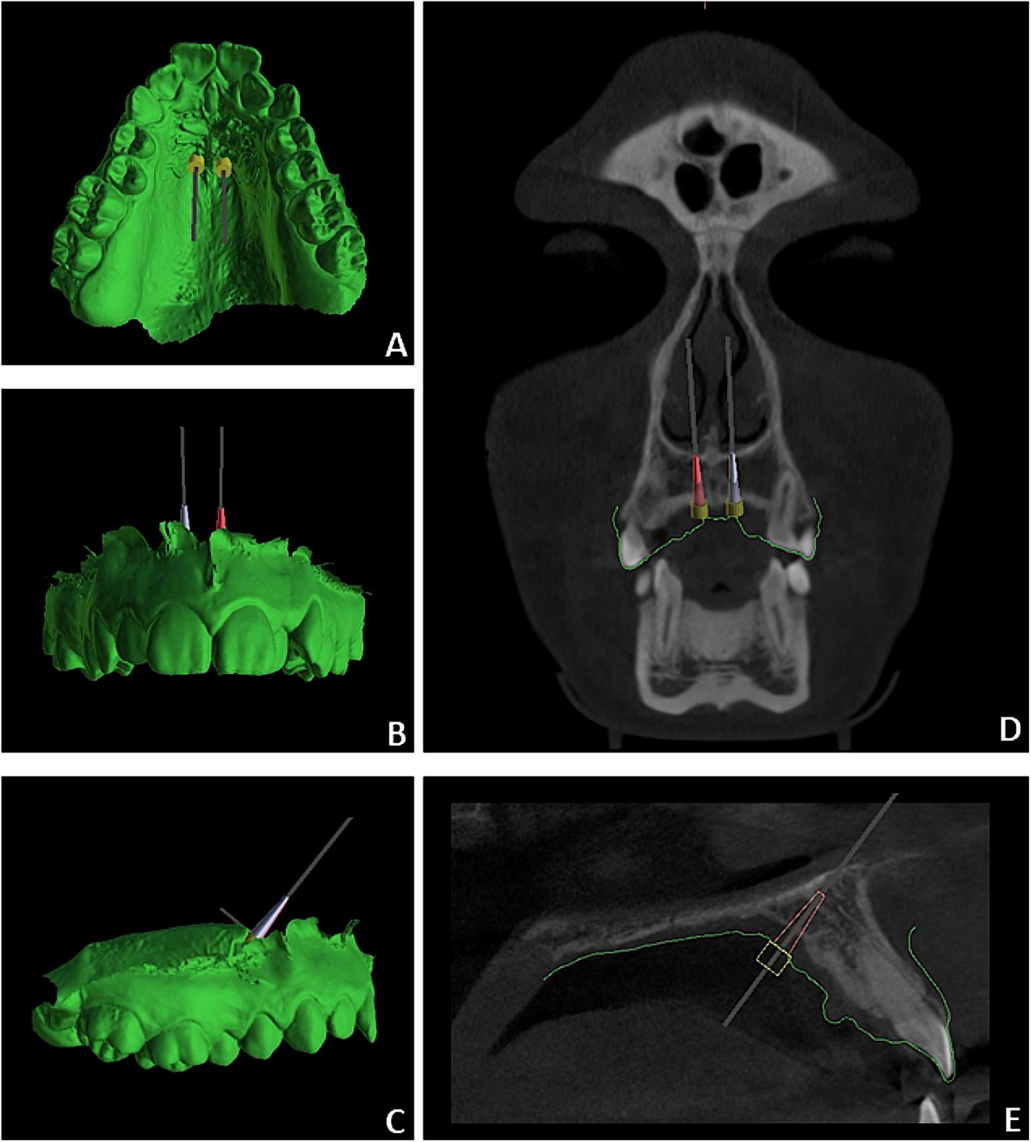
Virtual miniscrew insertion procedure performed using BB-PS **(A–C)** and CBCT-PS **(D,E)**.

In the first planning stage, the CBCT-derived STL file was hidden, and two miniscrews were positioned in the paramedian region according to established clinical guidelines derived from intraoral examination and model analysis. Miniscrews were placed at the level of the third palatal rugae, approximately 3 mm lateral to the palatal raphe, with an inclination that ensured parallelism between the lower surface of the abutment and the palatal mucosa (approximately 90°) (9, 17, 24). The miniscrew position was then refined to maintain parallelism, appropriate inclination relative to the occlusal plane, and minimal interference with the palatal slope mucosa. This workflow, performed without radiological data, was defined as the bone-blinded planning system (BB-PS) (Figures 1A–C). In the second stage, the CBCT-derived STL file was displayed, and miniscrew positioning was optimized by incorporating radiological information, including bone availability, insertion depth, mucosal thickness, and distances from incisor roots and the nasopalatine duct. This workflow was defined as the CBCT-based planning system (CBCT-PS) (Figures 1D,E) (24, 25). At the end of each planning stage, the maxillary IOS and the virtually placed miniscrews were merged and exported as a single STL file for the BB-PS (File A) and CBCT-PS (File B), respectively.

### 2.3 Digital analysis of miniscrews position

**Figure 2:**
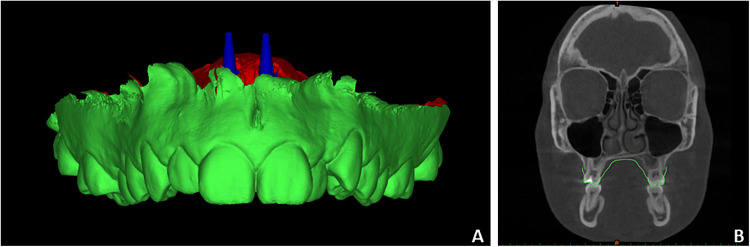
Example of single.stl file generated merging IOS and equivalent miniscrews **(A)** and imported into mimics software with CBCT scans **(B)**.

**Figure 3:**
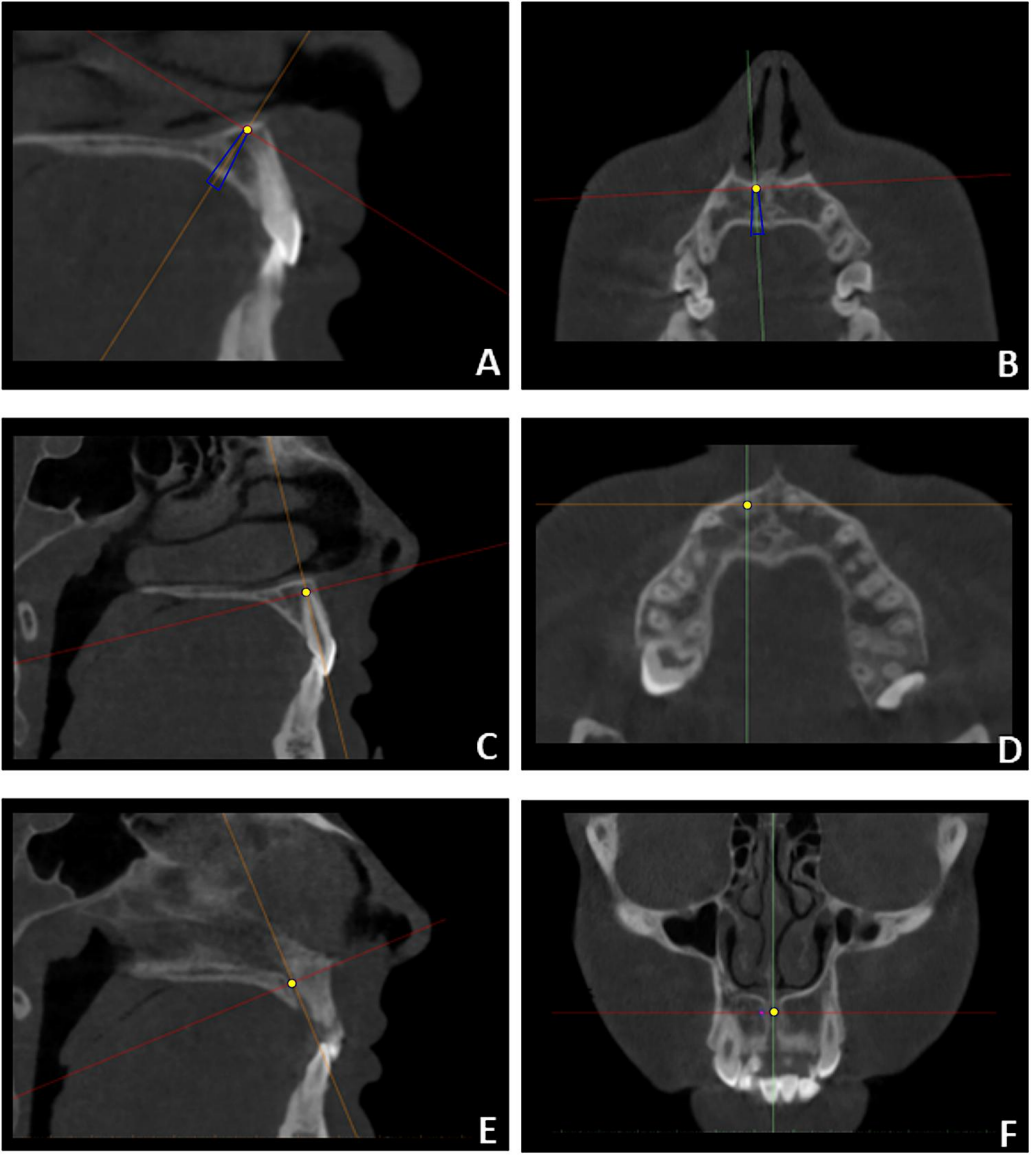
CBCT orientation and identification of landmarks used for the analysis: miniscrew's tip **(A,B)**, incisor's apex **(C,D)** and the minimum distance between the miniscrew and the nasopalatine duct (NP) **(E,F)**. These landmarks were retrieved after orientation of the *y*-axis respectively along central axis of the miniscrew **(A,B)**, the central axis of each maxillary incisor **(C,D)** and the axis of the nasopalatine duct **(E,F)**.

Files A and B, together with the CBCT datasets, were imported into Mimics software (Mimics 21.0; Materialise, Leuven, Belgium) to perform linear measurements assessing the proximity of the planned miniscrews to adjacent anatomical structures (Figures 2A,B). Although linear measurements can be obtained in Blue Sky Plan*, Mimics was selected for its superior capability to identify anatomical and miniscrew landmarks using multiplanar reconstructions. CBCT scans were temporarily reoriented to identify the reference landmarks for the analysis. The y- axis was aligned along the central axis of the miniscrew to mark the miniscrew's tip and centerline (Figures 3A,B). Afterward, the *y*-axis was aligned along the long axis of each maxillary incisor to identify and mark the root apex (Figures 3C,D). After landmark identification, the original CBCT orientation was restored, and the distances between the miniscrew tip and the maxillary right central (D11), right lateral (D12), left central (D21), and left lateral (D22) incisors were recorded. Finally, the *y*-axis was aligned with the nasopalatine duct, and the minimum distance between the miniscrew and the duct (NP distance) was measured on the axial slice passing through the miniscrew centerline (Figures 3E,F).

All digital planning procedures were performed by a single operator with 10 years of experience in skeletal anchorage and digital guided systems (A.L.G.). A second operator, with 5 years of experience in digital orthodontic applications, performed the measurements (V.R.). Data were collected in an Excel spreadsheet for statistical analysis. After a 4-week interval, all measurements were repeated under blinded conditions to assess intra- and inter-operator reliability.

### 2.4 Statistical analysis

A pilot study was conducted on 20 subjects (Group A = 10, Group B = 10) who satisfied the inclusion/exclusion criteria to evaluate sample size power. The analysis found that a sample size of 10 subjects per group achieves 97.8% power to detect a mean difference of 0.7 mm in the D11 distance between BB-PS and CBCT-PS with a known standard deviation of differences of 0.5 and with a significance level (a) of 0.05, using a 2-sided paired z-test. However, according to the inclusion criteria, we included 30 subjects per group, thereby increasing the robustness of the data. Descriptive statistics were carried out to examine linear differences between the miniscrew position and the sensitive structures (incisors' root apexes and naso-palatine duct). Shapiro–Wilk and Levene's tests were used to assess normality and equality of variances. Since all data showed normal distributions and equal variances, paired t-tests (*p* < 0.05) were used to compare measurements between BB-PS and CBCT-PS. Unpaired t-tests were used to compare the mean differences in the measurements recorded between Group A and Group B. The intraclass correlation coefficient (ICC; model = 2-way mixed effects, type = single measure, definition = absolute agreement) was used to calculate intra- and inter-examiner reliability for the superimposition and measurements workflow. Data were analyzed using SPSS® version 24 Statistics software (IBM Corporation, 1 New Orchard Road, Armonk, New York, USA) while sample size calculation was assessed using a web application (https://clincalc.com/stats/samplesize.aspx).

## 3 Results

No significant differences in age, sex distribution, or skeletal maturation stage were observed between the two groups, confirming sample homogeneity (Table 1). In Group A, no significant differences were found in the distances recorded between incisors and paramedian miniscrews planned with BB-PS and CBCT-PS methods (*p* > 0.05); the same findings were retrieved in relation to the distance between miniscrews and nasopalatine duct (*p* > 0.05) (Table 2). In Group B, significant differences were found in the distances recorded between incisors and paramedian miniscrews planned with BB-PS and CBCT-PS methods (*p* > 0.05); also, miniscrews planned with BB-PS were statistically closer to the nasopalatine duct compared to those scheduled after correction using CBCT (*p* < 0.05) (Table 3). Comparing data measurements between the two groups, statistical significance was found in the mean differences of the distances from the naso-palatine duct planned with the BB-PS and CBCT-PS methods (*p* < 0.05). Regarding measurement reliability, ICC tests showed no difference between the two readings, with excellent intra-observer (0.869–0.920) and inter-observer (0.812–0.857) correlations (Table 4).

**Table 2:** Group A (normal palate dimension): distances between miniscrews and sensible structures (incisors’ roots and naso-palatine duct) measured with BB-PS and CBCT-PS methods.

Outcome	Sides	Measure	Group A									
			BB-PS				CBCT-PS					
			Mean	SD	Min	Max	Mean	SD	Min	Max	Mean difference	*p* value*
TAD-Roots distance	Right TAD	D12	6.6	2.11	4.9	10.9	6.9	2.1	4.6	11.0	−0.3	*p* = 0.464
		D11	7.4	2.2	5.2	11.6	7.2	2.2	4.9	11.2	0.2	*p* = 0.061
	Left TAD	D21	7.6	2.1	5.4	11.5	7.5	2.0	4.4	11.3	0.1	*p* = 0.358
		D22	6.6	2.02	4.6	11.1	7.0	1.9	4.8	11.4	−0.4	*p* = 0.254
TAD-Nerve distance	Right TAD	DNP	3.5	1.4	1.0	5.7	3.8	1.3	1.8	6.2	−0.3**	*p* = 0.067
	Left TAD	DNP	3.6	1.3	1.6	5.8	4.0	0.8	2.9	5.6	−0.4**	*p* = 0.171

D12, D11, D21, D22 = distance between miniscrew and incisor's root; DNP = distance between miniscrew tip and naso-palatine nerve.

* *p* value based on Paired Student t test and set at *p* < 0.05.

** *p* value based on Independent Student t test and set at *p* < 0.05 for inter-group comparisons.

**Table 3:** Group B (maxillary constriction): distances between miniscrews and sensible structures (incisors’ roots and naso-palatine duct) measured with BB-PS and CBCT-PS methods.

Outcome	Sides	Measure	Group B									
			BB-PS				CBCT-PS					
			Mean	SD	Min	Max	Mean	SD	Min	Max	Mean difference	*p* value*
TAD-Roots distance	Right TAD	D12	7.0	2.0	3.7	9.8	6.4	1.9	3.1	8.9	0.6	*p* < 0.001
		D11	8.0	2.0	5.1	10.6	8.6	1.9	5.4	11.1	−0.6	*p* < 0.001
	Left TAD	D21	8.2	2.1	4.6	10.6	8.7	2.1	5.0	11.6	−0.5	*p* < 0.001
		D22	7.6	2.2	3.8	12.2	7.1	1.8	3.1	10.0	0.5	*p* < 0.001
TAD-Nerve distance	Right TAD	DNP	2.6	1.1	0	4.5	3.5	0.9	1.8	5.6	−0.9**	*p* = 0.001
	Left TAD	DNP	2.3	1.3	0	4.2	3.3	0.6	2.0	5.0	−1.0**	*p* < 0.001

D12, D11, D21, D22 = distance between miniscrew and incisor's root; DNP = distance between miniscrew tip and naso-palatine nerve.

* *p* value based on Paired Student t test and set at *p* < 0.05.

** *p* value based on Independent Student t test and set at *p* < 0.05 for inter-group comparisons.

**Table 4:** Intra-operator and inter-operator reliability for digiital workflow and measurements performed for group A and group B.

Outcome	Sides	Measure	Intra-operator			
			BB-PS		CBCT-PS	
			ICC	Agreement	ICC	Agreement
TAD-Roots distance	Right TAD	D12	0.888	Near complete agreement	0.920	Near complete agreement
		D11	0.877	Near complete agreement	0.909	Near complete agreement
	Left TAD	D21	0.893	Near complete agreement	0.898	Near complete agreement
		D22	0.869	Near complete agreement	0.911	Near complete agreement
Outcome	Sides	Measure	INTRA-OPERATOR			
			ICC	AGREEMENT	ICC	AGREEMENT
TAD-Nerve distance	Right TAD	DNP	0.812	Near complete agreement	0.845	Near complete agreement
	Left TAD	DNP	0.833	Near complete agreement	0.857	Near complete agreement

D12, D11, D21, D22 = distance between miniscrew and incisor's root; DNP = distance between miniscrew tip and naso-palatine nerve.

Analysis performed via Intraclass correlation coefficient (ICC); model = 2-way mixed effects, type = single measure, definition = absolute agreement).

## 4 Discussion

To the best of our knowledge, this is the first study to simulate the risk of root and neurovascular bundle damage during miniscrew insertion in the anterior palate without radiographic data. Ideally, such information would be obtainable after registration of the post-insertion intra-oral scan (with scanbodies) with the pre-insertion CBCT (26); however, when opting for direct insertion, clinicians do not request CBCT examination. Consequently, simulation studies are the only means to approximate this risk. Although the bone-blinded planning system (BB-PS) cannot replicate the clinical procedure of direct insertion, in which manual skills play a critical role, both approaches require careful assessment of palatal morphology on digital or analog models in the absence of radiological data. In the specific case of direct insertion, this need is further emphasized by the limited direct visualization during the surgical procedure.

A major strength of the present study is the comparative analysis between subjects with normal transverse maxillary dimensions (Group A) and those with maxillary constriction (Group B). In this context, the present findings, although derived from a digital simulation, have relevant clinical implications, particularly considering the increasing use of skeletal anchorage in the paramedian region for palatal expansion in post-adolescent patients (27).

In both groups, BB-PS planning resulted in a safety distance between miniscrews and maxillary incisors, ranging from approximately 6 to 8 mm. This finding supports the safety of the procedure with respect to root damage and is consistent with previous studies using guided systems based on lateral cephalograms (16) and comparisons between direct insertion and guided approaches (10). This outcome may be explained by the operator's tendency to follow the inclination of the palatal mucosal profile, thereby maintaining a miniscrew trajectory that preserves an adequate distance from root apices even in the absence of radiographic guidance. However, this explanation does not account for positional alterations (e.g., palatal tooth ectopia or anomalous palatal root torque of the upper incisor) or morphological anomalies (e.g., dilacerated dental elements with palatal apex version) that must be identified during the diagnostic process and require in-depth analysis with CBCT.

When BB-PS was used, miniscrews were positioned closer to the nasopalatine duct in Group B (approximately 2 mm) than in Group A (approximately 3 mm). In some cases, the distance was critical, approaching 1 mm. In two simulated cases, the lateral wall of the nasopalatine duct was even in contact with the medial surface of the miniscrew at its central portion, where the screw diameter is greater than at the tip. For this reason, it was often necessary to adjust the screw positions using CBCT-PS, moving the miniscrews away from the palatal raphe, as suggested by the significant differences recorded between BB-PS and CBCT-PS. The reasonable explanation of these findings is that, in patients with a narrow palate, the operator tended to position the screws closer to the raphe due to limited transverse dimensions. This observation is clinically relevant, considering the substantial anatomical variability of the nasopalatine canal. A Y-shaped configuration is reported in approximately 60%–65% of cases, whereas parallel canals are observed in only 10%–15% (28, 29). Future studies with larger samples should therefore assess miniscrew safety not only in relation to transverse palatal dimensions but also to nasopalatine canal morphology. Clinically, these findings support the recommendation to use CBCT and guided systems in patients with narrow palates, especially given the higher risk of inclination errors during direct insertion (10). Moreover, minor deviations in inclination may also occur with guided systems compared to the planned position (8, 30).

Another factor contributing to the critical proximity between BB-PS miniscrews and the nasopalatine duct may be the operator's attempt to preserve sufficient space between the intraoral miniscrew head and the lateral palatal slope of a constricted maxilla. Clinically, an adequate distance between the miniscrew head and the lateral palatal slope is critical to avoid the traumatic impact of the connector on the mucosa (Supplementary Figure S1) (13). The pressure generated by such contact may also induce tensile forces that could affect miniscrew stability during expansion, although evidence on this aspect is currently lacking. Despite the low vascular density of the anterior palate and the generally minimal risk of iatrogenic injury during miniscrew insertion (21, 31), avoiding proximity to the nasopalatine duct should take precedence over minimizing the distance between the miniscrew head and the lateral palatal slope. In patients requiring maxillary expansion, this proximity should therefore be avoided (Supplementary Figures S2a–c). In such cases, increasing the inter-miniscrew distance (Supplementary Figures S2d–f) and requesting customization of the connector may reduce resistance and improve appliance fit. Minor degrees of inclination may also be tolerated without significantly compromising miniscrew parallelism (Supplementary Figures S2g–i). Finally, asymmetry of the nasopalatine duct has been reported (32), potentially increasing the risk of unilateral miniscrew impingement. Based on these considerations, CBCT should be recommended when miniscrews are inserted in the paramedian region in patients with limited transverse palatal dimensions.

Based on our results, it may be inferred that, in patients without clinical signs of skeletal maxillary discrepancy, knowledge of the T-Zone map, combined with careful analysis of anterior palatal morphology may be sufficient to ensure safe paramedian miniscrew placement. However, this conclusion should be interpreted with caution to avoid the misconception that radiographic examinations or guided systems are unnecessary in patients with apparently adequate palatal dimensions. For instance, CBCT- or cephalogram-based guided systems remain essential when bicortical anchorage is required (33).

### 4.1 Limitations

- First, its external validity is restricted by the relatively small sample size, which did not allow inclusion of a broad spectrum of interindividual anatomical variability of the anterior palate. In addition, the use of a virtual simulation design represents an inherent limitation when extrapolating the results to clinical practice.
- Second, patients were not stratified according to sagittal skeletal pattern or growth pattern, which limits the generalizability of the findings. Future investigations should therefore compare the risk of root injury in subjects with skeletal Class III relationships or maxillary retrusion with those presenting normal anteroposterior growth, as reduced bone availability in the paramedian region may be expected in the former group.
- A further limitation concerns the exclusive use of a single miniscrew length (9 mm). However, this should not be considered a major drawback for several reasons: 1) the study did not aim to statistically assess direct impaction events between miniscrews and adjacent anatomical structures, an analysis that would have required the use of multiple screw lengths; 2) because the investigation focused on linear distance measurements between the miniscrew tip and sensitive anatomical structures, the reported values inherently provide clinicians with information relevant for selecting alternative screw lengths when needed; 3) the decision to model all simulations using a 9-mm miniscrew is consistent with a conservative clinical approach to miniscrew placement in the absence of three-dimensional radiological imaging. This reflects the prudent clinicians’ approach who prefer selecting the shortest miniscrew length among those proven reliable in the literature.

## 5 Conclusion

Within the limitations of the present study, virtual miniscrew planning without radiographic information (BB-PS) resulted in a safe distance from sensitive anatomical structures, including incisor roots and the nasopalatine duct, in subjects with normal transverse palatal dimensions. In contrast, the risk of nasopalatine duct injury was significantly higher in subjects with maxillary constriction, supporting the recommendation for CBCT-based planning in these patients.

## Data Availability

The raw data supporting the conclusions of this article will be made available by the authors, without undue reservation.

## Supplementary material

Supplementary Figure S1 Example of the critical distance maintained by the operator between the miniscrew head and the palatal slope.

Supplementary Figure S2 Example of miniscrew insertion in presence of narrowed palate: BB-PS = miniscrews placed closer to the palatine raphe (a), with adequate space between the abutment and the palate slope (b) and critical position in relation to the naso-palatine duct (c); CBCT-PS = miniscrews placed at greater distance to the palatine raphe (d), with compression of the abutment (miniscrews’ head) to the palate slope (e) and adequate position in relation to the naso-palatine duct (f). Miniscrews can be inclined to avoid the impact of the abutment to the palatal mucosa the, however this option affects the parallelism between miniscrews (g–i).
