# Supplementary figures and images for: Simulated risk of root and neurovascular bundle damage during miniscrew insertion in the anterior palate without radiological data
Source: Front Dent Med. 2026 Mar 9;7:1729079. doi: 10.3389/fdmed.2026.1729079 (PMC13006589; doi:10.3389/fdmed.2026.1729079)

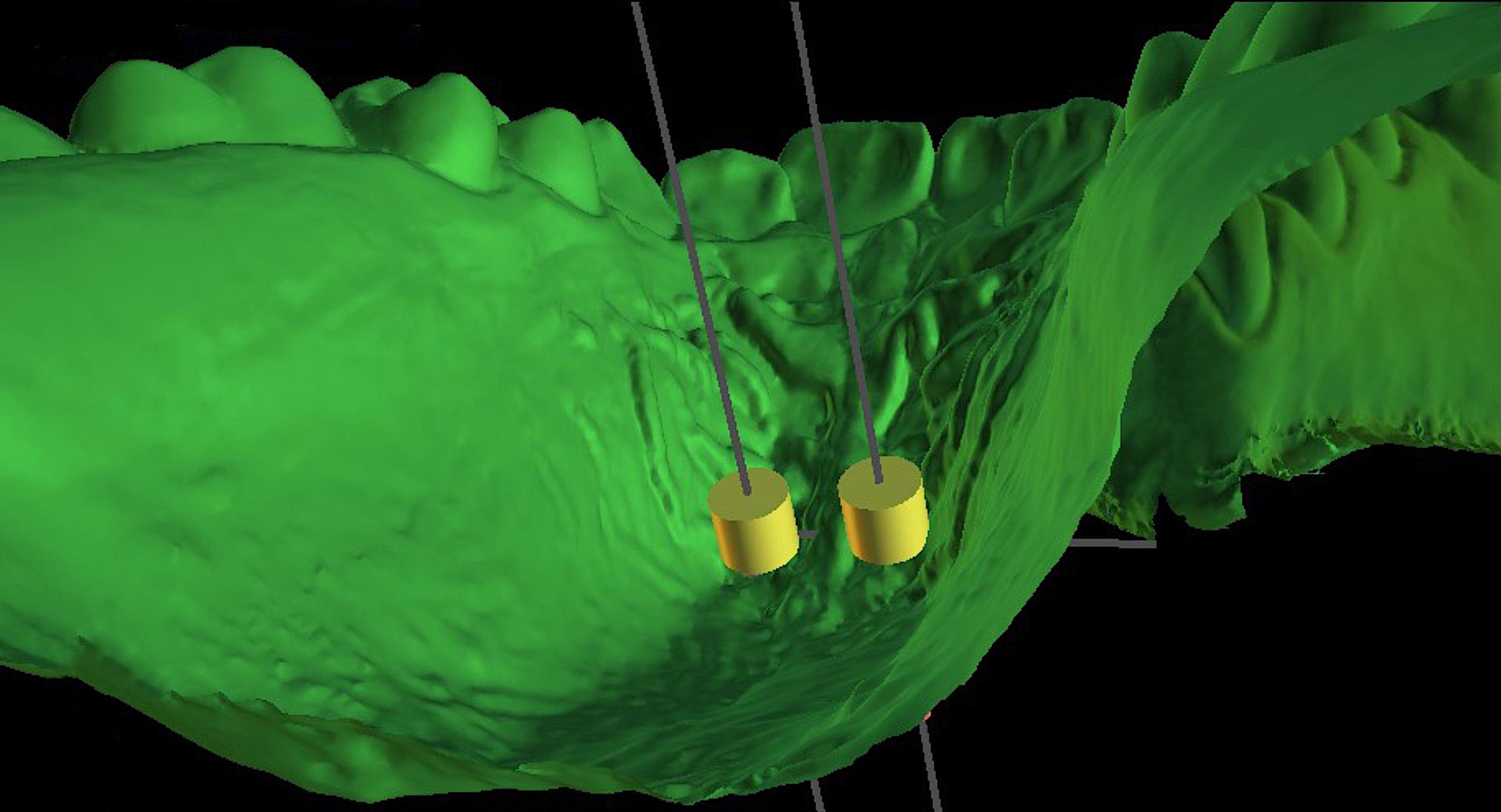

Supplement: Supplementary Figure S1 — Example of the critical distance maintained by the operator between the miniscrew head and the palatal slope. [file Image1.tiff]

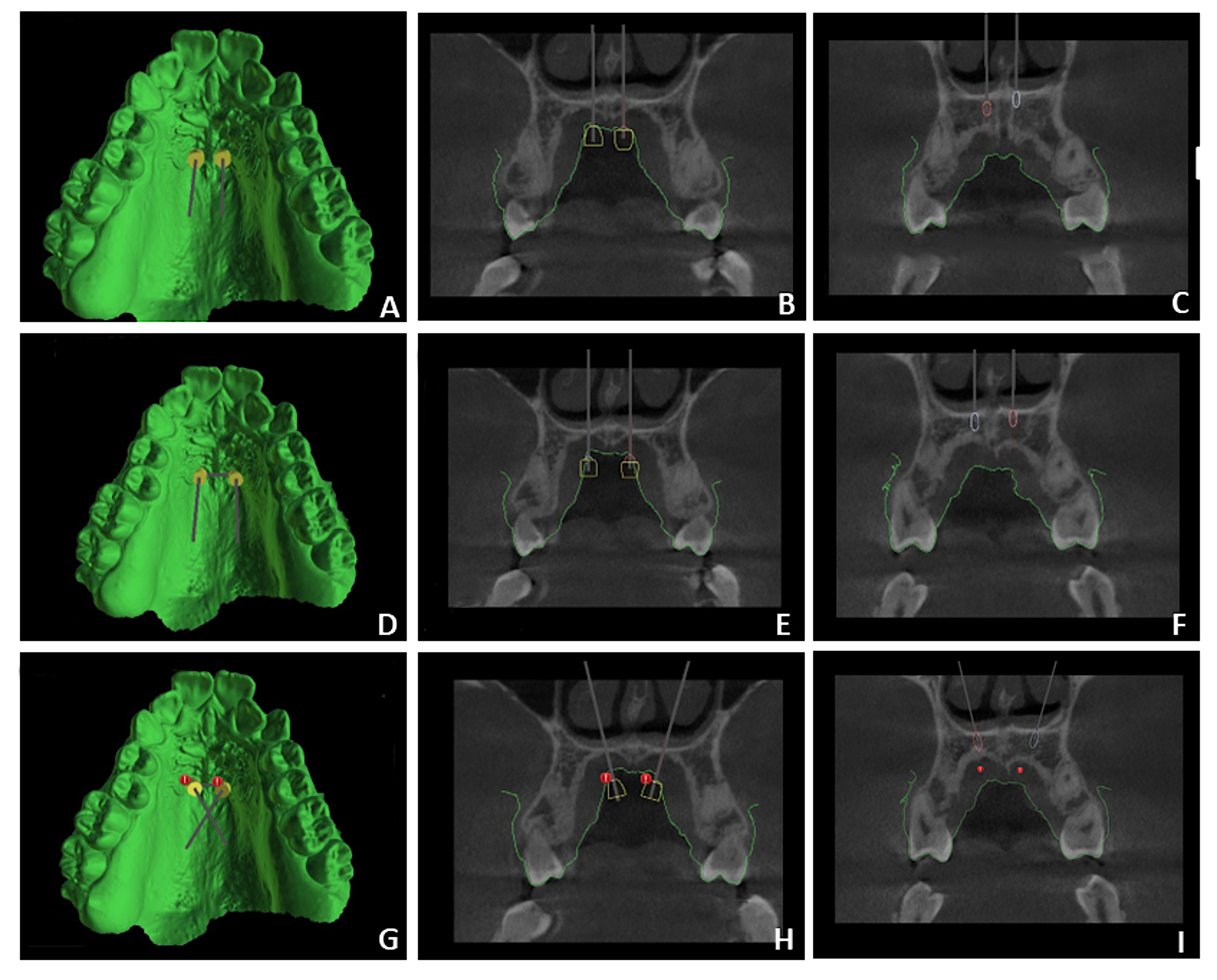

Supplement: Supplementary Figure S2 — Example of miniscrew insertion in presence of narrowed palate: BB-PS = miniscrews placed closer to the palatine raphe (a), with adequate space between the abutment and the palate slope (b) and critical position in relation to the naso-palatine duct (c); CBCT-PS = miniscrews placed at greater distance to the palatine raphe (d), with compression of the abutment (miniscrews’ head) to the palate slope (e) and adequate position in relation to the naso-palatine duct (f). Miniscrews can be inclined to avoid the impact of the abutment to the palatal mucosa the, however this option affects the parallelism between miniscrews (g–i). [file Image2.tiff]
